# Sex Differences in Outcome of Aneurysmal Subarachnoid Hemorrhage and Its Relation to Postoperative Cerebral Ischemia

**DOI:** 10.1007/s12028-024-02028-9

**Published:** 2024-07-01

**Authors:** Cheng Yang, Zenan Zhao, Biao Yang, Kaishan Wang, Gang Zhu, Hongping Miao

**Affiliations:** 1 Department of Neurosurgery, Southwest Hospital, Army Medical University, Chongqing, 400038 China; 2 Department of Neurosurgery, Chongqing Western Hospital, Chongqing, 400052 China; 3 Department of Neurosurgery, Dazu Hospital of Chongqing Medical University, Chongqing, 402360 China

**Keywords:** Subarachnoid hemorrhage, Cerebral infarction, Aneurysm, Sex characteristics

## Abstract

**Background:**

Whether there is a sex difference in the outcome of aneurysmal subarachnoid hemorrhage (aSAH) remains controversial, and clarifying the role of women in postoperative cerebral ischemic events can help us to understand its relationship with poor prognosis. Therefore, the purpose of this study was to elucidate the relationship between the three aspects of sex differences, postoperative cerebral ischemia, and poor prognosis after aSAH.

**Methods:**

A total of 472 patients admitted within 72 h after aSAH between January 2018 and December 2022 were included. We systematically analyzed the characteristics of sex differences in aSAH and explored the relationship between delayed cerebral ischemia (DCI), surgery-related cerebral infarction (SRCI), and poor prognosis (modified Rankin Scale > 2).

**Results:**

Compared with women, men were in worse condition and had more intracerebral hematoma (*p* = 0.001) on admission, whereas women were older (*p* < 0.001) and had more multiple aneurysms (*p* = 0.002). During hospitalization, men were more likely to experience emergency intubation (*p* = 0.036) and tracheotomy (*p* = 0.013). Women achieved functional independence at discharge at a similar rate to men (*p* = 0.394). Among postoperative complications, the incidence of DCI (22% vs. 12%, *p* = 0.01) and urinary tract infection (*p* = 0.022) was significantly higher in women. After adjusting for age, multivariable regression analysis showed that hypertension (odds ratio [OR] 2.139, 95% confidence interval [CI] 1.027–4.457), preoperative rerupture (OR 12.240, 95% CI 1.491–100.458), pulmonary infection (OR 2.297, 95% CI 1.070–4.930), external ventricular drainage placement (OR 4.382, 95% CI 1.550–12.390), bacteremia (OR 14.943, 95% CI 1.412–158.117), SRCI (OR 8.588, 95% CI 4.092–18.023), venous thrombosis (OR 5.283, 95% CI 1.859–15.013), higher modified Fisher grades (*p* = 0.003), and Hunt–Hess grades (*p* = 0.035) were associated with poor prognosis, whereas DCI (OR 1.394, 95% CI 0.591–3.292) was not an independent risk factor for poor prognosis. The proportion of patients who fully recovered from cerebral ischemia was higher in the DCI group (*p* < 0.001) compared with the SRCI group, and more patients were discharged with modified Rankin Scale > 2 in the SRCI group (*p* = 0.005).

**Conclusions:**

Women have a higher incidence of DCI, but there is no sex difference in outcomes after aSAH, and poor prognosis is associated with worse admission condition and perioperative complications. SRCI is a strong independent risk factor for poor prognosis, whereas DCI is not.

## Introduction

Aneurysmal subarachnoid hemorrhage (aSAH) is the abnormal accumulation of blood components in the subarachnoid space after aneurysm rupture, which accounts for 85% of all spontaneous SAH events [1]. Early treatment of ruptured aneurysm reduces the risk of rebleeding [2], but early brain damage after hemorrhage and secondary complications, such as hydrocephalus and delayed cerebral ischemia (DCI), leave survivors with a reduced quality of life in the long term [3].

In the face of this catastrophe, sex has not been treated fairly, and women have been shown to have more characteristics of aneurysm and SAH [4]. Compared with men, women have a higher rate of intracranial aneurysm and are more likely to experience aneurysm growth [5, 6], and women have a higher risk of aneurysm rupture than men, with a hazard ratio of 1.39 [7]. This difference is even more pronounced in all patients with aSAH, where the proportion of women is approximately twice that of men [8–11]. Although the present literature summarizes the basic characteristics of ruptured aneurysms by sex [4], the questions of whether changes in women’s hormone levels lead to a higher risk of aSAH and whether sex affects treatment outcomes have not been fully elucidated, and sex differences still deserve further study.

According to a recent study, the worst prognosis of female patients after aSAH cannot be rationalized by the intensity of treatment and management [12], and other studies have suggested that the poor outcome can be attributed to more complications in women, especially vasospasm and cerebral ischemia [10, 11, 13–15]. However, these finding have not been confirmed in other studies [16, 17]. Therefore, in this study, we analyzed differences in the treatment and prognosis of ruptured aneurysms based on sex and explained the reasons in terms of clinical characteristics and perioperative complications.

## Methods

### Data Availability Statement

This study was approved by the Ethics Committee of the First Affiliated Hospital of Army Medical University, and informed consent was exempt. Data supporting this study are available from the corresponding author on reasonable request.

### Patient Selection

We conducted a retrospective study of patients aSAH admitted to the Department of Neurosurgery of the First Affiliated Hospital of the Army Medical University between January 2018 and December 2022. The terms “aneurysm” and “subarachnoid hemorrhage” were used to search for patients admitted to the neurosurgery department from 2018 to 2022 in the electronic medical record system. Inclusion criteria were as follows: (1) patients older than 18 years and admitted within 72 h after aSAH, (2) aSAH diagnosed clinically by imaging or lumbar puncture, and (3) surgical aneurysm repair (clip or endovascular) performed during hospitalization. Exclusion criteria were as follows: (1) other causes of SAH, such as arteriovenous malformations, trauma, or hypertensive cerebral hemorrhage; (2) any surgical treatment for ruptured aneurysm in other hospitals prior to admission; (3) severe physically disability; (4) patients with incomplete clinical and imaging data; and (5) nonspontaneous aneurysm rupture.

### Data Selection and Definition

Data at admission included the following: age, sex, blood type, Glasgow Coma Scale (GCS) score, World Federation of Neurological Surgeons (WFNS) grade, Hunt–Hess grade, modified Fisher grade [18], menstrual history, hypertension, diabetes, hyperlipidemia, smoking, drinking, location and size of aneurysm, multiple aneurysms, intracerebral hematoma, and acute hydrocephalus. Treatment details included the following: surgical approach (clip or endovascular), stent use, external ventricular drainage (EVD) placement, and lumbar drainage. Perioperative complications included the following: preoperative rerupture, intraoperative rerupture, DCI, surgery-related cerebral infarction (SRCI), urinary tract infection, intracranial infection, pulmonary infection, venous thrombosis, and bacteremia. Airway management included emergency intubation and tracheotomy. Indicators at discharge included the GCS score and modified Rankin Scale (mRS).

The timing of menopause was obtained through our electronic medical record system. Typically, the patient’s medical history information was taken by the physician on admission, and the menstrual history was provided by the awake patient herself or family members if the patient was unconscious. Data were recorded as missing when neither the patient nor the family could provide a menstrual history. Accurate time (year) of menopause could not be obtained in some cases and was also recorded as missing data. Acute hydrocephalus was defined as an Evan’s index > 0.3 on the admission head computed tomography (CT) scan. Emergency intubation was defined as rescue tracheal intubation during hospitalization, except for general anesthesia procedures. Preoperative rerupture was defined as a deterioration in consciousness that occurred after admission and was confirmed by preoperative CT or by craniotomy with mismatched (increased) hemorrhage compared to the first CT scan after admission. Venous thrombosis was defined as ultrasound-detected thrombosis in the jugular and extremity veins. Heparin was not routinely used prophylactically in patients with aSAH at our institution. For those at high risk of deep vein thrombosis, early limb rehabilitation (by specialized nurse practitioners) was provided. If venous thrombosis was detected by early ultrasound, heparin therapy might be used after assessing the risk of bleeding. Functional independence and poor prognosis were defined as mRS ≤ 2 and mRS > 2 at discharge, respectively. SRCI was defined as neurologic deterioration or asymptomatic cerebral infarction occurring in the early postoperative period (usually within 24 h and no longer than 48 h postoperatively), with new infarction observable on imaging, and could be inferred to be related to surgery from surgical records or postoperative CT, excluding other factors, such as cerebral hemorrhage, hydrocephalus, and use of sedation. Referring to previous criteria [19, 20], DCI was defined as the occurrence of focal neurologic injury (hemiparesis, aphasia, apraxia, hemianopia, or neglect) or a reduction in the GCS score of at least 2 points (total score or one of its individual components) within 21 days after aSAH. This condition lasted more than 1 h, was not evident after aneurysm occlusion, and could not be attributed to other causes by clinical assessment. In addition, after excluding the effects of SRCI, cerebral infarction due to atrial fibrillation, and severe cardiopulmonary failure, we considered new-onset cerebral infarctions observed on postoperative imaging as DCI, and the progression of SRCI on CT should not be considered as DCI.

### Postoperative Imaging Program

Our neurosurgical care unit is equipped with proximity head CT, which enhances the probability that deterioration will be captured by imaging at an early stage. Patients typically have their head CT scan reviewed within 12 and 48 h after surgery, which is more frequent in patients with a poor clinical condition, and additional CT and/or magnetic resonance imaging during hospitalization is scheduled according to the patient’s condition.

### Statistical Analysis

Statistical analysis was performed using SPSS (version 25). Continuous variables were expressed as mean ± SD or median (interquartile range) according to whether they were normally distributed, and categorical variables were expressed as percentages and divided into two groups by sex for between-group comparison. Continuous variables with a normal distribution were compared using Student’s *t*-test, otherwise the Mann–Whitney *U*-test was performed. The χ^2^ test or Fisher’s exact test was used for comparison of categorical variables. Factors with a *p* value < 0.1 in the univariate analysis were incorporated in the regression model, including the following: age, hypertension, smoke, hyperlipidemia, modified Fisher grade, Hunt–Hess grade, multiple aneurysms, intracerebral hematoma, preoperative rerupture, clip, EVD placement, pulmonary infection, bacteremia, SRCI, DCI, and venous thrombosis. To describe the age distribution, time from menopause to aneurysm rupture, and timing of DCI, histograms were plotted. Multivariable analysis was performed using binary logistic regression with mRS > 2 at discharge as poor prognosis. Finally, the DCI and SRCI were compared using the χ^2^ test, and *p* < 0.05 was considered statistically significant in the study.

## Results

### Basic Characteristic

**Fig. 1:**
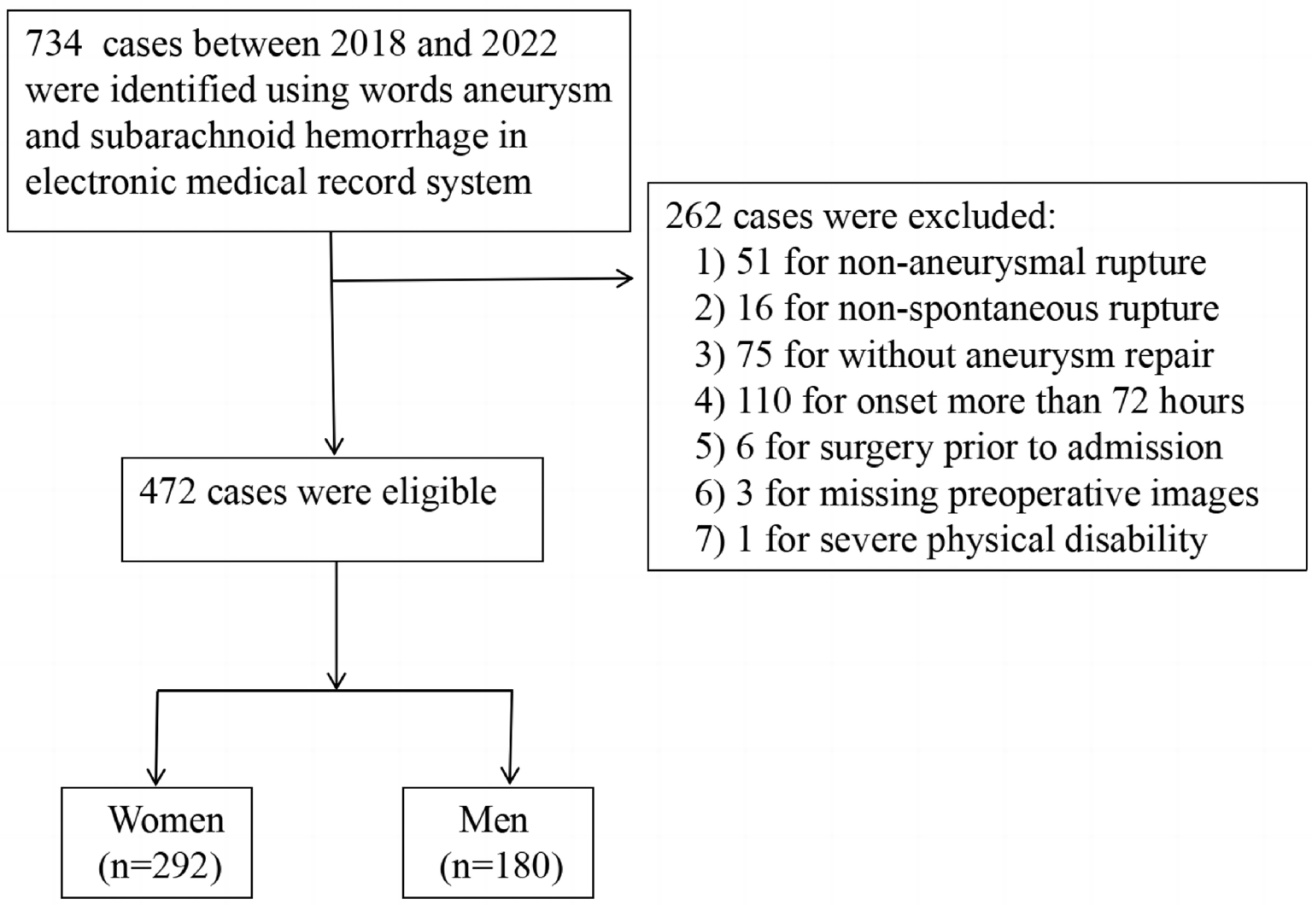
Flowchart for case screening

As shown in Fig. 1, we identified 734 cases between 2018 and 2022 using the terms “aneurysm” and “subarachnoid hemorrhage” in the electronic medical record system. After excluding 262 cases through inclusion and exclusion criteria, 472 patients were finally included (292 women and 180 men).

**Fig. 2:**
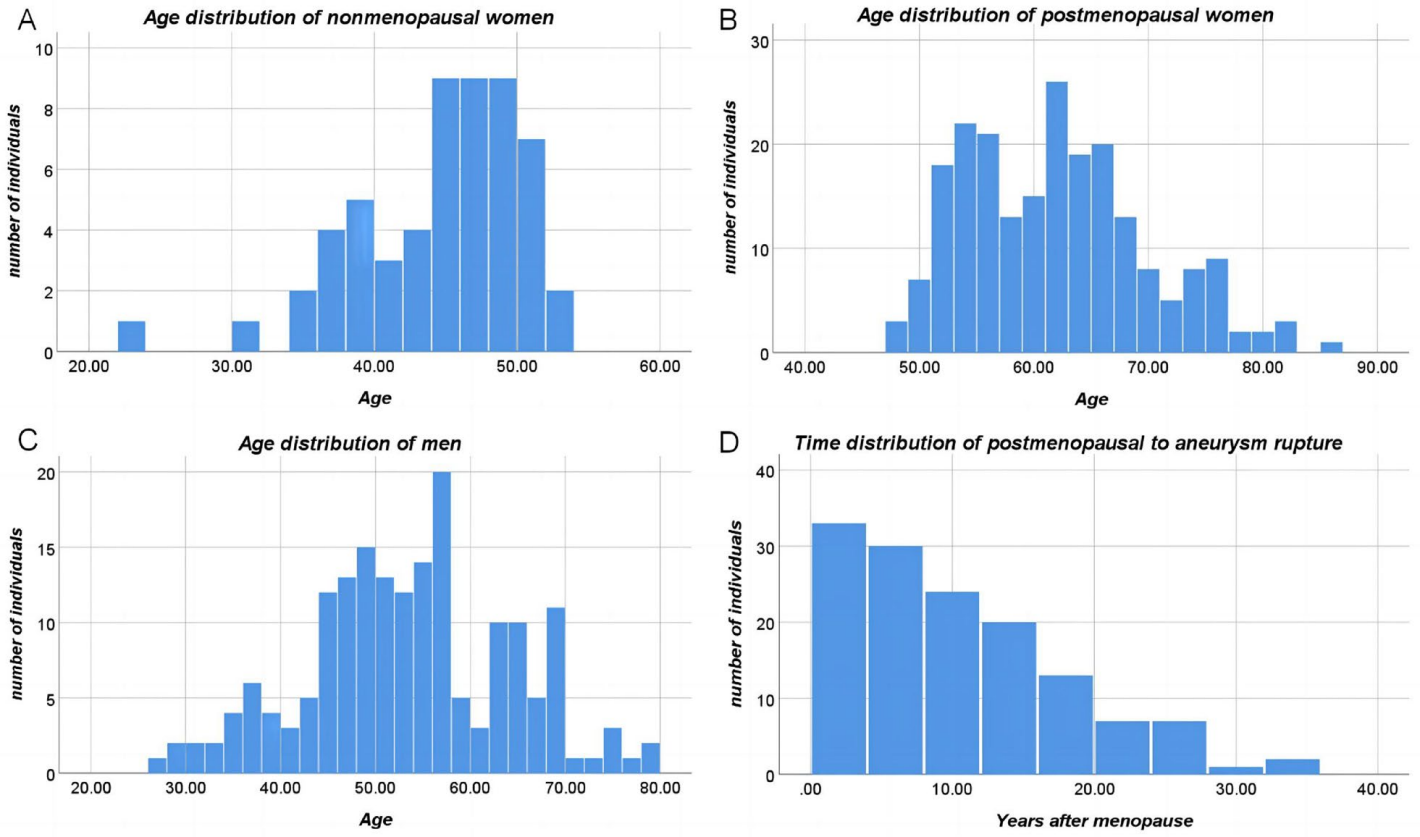
Age distribution of patients with aneurysmal subarachnoid hemorrhage (aSAH) by sex. Twenty-one women from whom we could not obtain a history of menstruation were excluded. **a**, **b**, and **c** represent the age distribution of 56 nonmenopausal women, 215 postmenopausal women, and 180 men, respectively. Excluding 78 women who lacked a specific time of menopause, the time experienced from menopause to aneurysm rupture was recorded in 137 women (**d**)

In our cohort of patients with aSAH, 61.8% were women, and 79% of the ruptured aneurysms in women occurred after menopause (Table 1). The median age at onset was 53 years for men and 56 years for women, with a significantly higher average age for women. Menstrual information was recorded in 271 women, and the time from menopause to aneurysm rupture was available in 137 cases. Histograms (Fig. 2) showed that the age peak for nonmenopausal women and postmenopausal women was concentrated between the ages of 45 and 52 and 52 and 69, respectively. The highest number of aneurysm ruptures occurred in the first 4 years after menopause, with a gradual decline in each subsequent 4-year period. The peak age at onset in men was mainly between 45 and 58 years. Compared with women, men had worse clinical status and higher bleeding grades (worse GCS score, modified Fisher grade, WFNS grade) on admission, and Hunt–Hess grades also showed a tendency to be worse in men but did not reach a statistical difference. Multiple aneurysms were more common in women (*p* = 0.002), whereas men had a higher rate of intracerebral hematoma on the admission CT scan (*p* = 0.001). A total of 4% (20 of 472) of patients experienced preoperative aneurysm rerupture, with no significant sex difference. In women, 47% (137 of 292) of ruptured aneurysms were located in the C7 (posterior communicating segment) of the internal carotid artery, followed by the anterior communicating artery (24%) and the middle cerebral artery (12%). In contrast, anterior communicating artery aneurysms were most common in men (44%), with C7 segment and middle cerebral artery aneurysms accounting for similar proportions (22% and 18%, respectively).

**Table 1:** Comparison of general information at admission of patients by sex

Variable	All (*N* = 472)	Women (*n* = 292)	Men (*n* = 180)	*P* value
Age, y, median (IQR)	56 (49–63)	56 (51–64)	53 (46–61)	< 0.001*
Menopause, *n* (%)	NA	215/271(79%)	NA	
Missing data		21		
Hypertension, *n* (%)	242 (51%)	156 (53%)	86 (48%)	0.233
Diabetes, *n* (%)	27 (6%)	19 (7%)	8 (4%)	0.349
Hyperlipidemia, *n* (%)	190/411 (46%)	119/253 (47%)	70/158 (44%)	0.589
Missing data	61	39	22	
Smoker, *n* (%)	117 (25%)	7 (2%)	110 (61%)	< 0.001*
Drinker, *n* (%)	89 (19%)	8 (3%)	81 (45%)	< 0.001*
GCS, median (IQR)	15 (14–15)	15 (14–15)	15 (12–15)	0.027*
WFNS, median (IQR)	1 (1–2)	1 (1–2)	1 (1–4)	0.017*
Modified Fisher, median (IQR)	2 (2–4)	2 (2–3)	2 (2–4)	0.012*
Hunt–Hess, median (IQR)	2 (1–3)	2 (1–3)	2 (1–3)	0.066
Multiple aneurysms, *n* (%)	133 (28%)	97(33%)	36(20%)	0.002*
Intracerebral hematoma, *n* (%)	102 (22%)	48 (16%)	54 (30%)	0.001*
Acute hydrocephalus, *n* (%)	60 (13%)	35 (12%)	25 (14%)	0.547
Preoperative rerupture, *n* (%)	20 (4%)	10 (3%)	10 (6%)	0.264
Blood type, *n* (%)				0.173
A	151/462 (33%)	95/286 (33%)	56/176 (32%)	
B	114/462 (25%)	61/286 (21%)	53/176 (30%)	
AB	41/462 (9%)	28/286 (10%)	13/176 (7%)	
O	156/462 (34%)	102/286 (36%)	54/176 (31%)	
Missing data	10	6	4	
Aneurysm location, *n* (%)				< 0.001*
ICA (C6)	26 (6%)	22 (8%)	4 (2%)	
ICA (C7)	177 (38%)	137 (47%)	40 (22%)	
ACA	20 (4%)	11 (4%)	9 (5%)	
ACoA	148 (31%)	69 (24%)	79 (44%)	
MCA	67 (14%)	34 (12%)	33 (18%)	
PC	34 (7%)	19 (7%)	15 (8%)	
Treatment, *n* (%)				0.059
Clip	169 (36%)	95 (33%)	74 (41%)	
Endovascular	303 (64%)	197 (67%)	106 (59%)	
During surgery, *n* (%)				
Stent assist	246/303 (81%)	165/197 (84%)	81/106 (76%)	0.015*
Intraoperative rerupture	13 (3%)	10 (3%)	3 (2%)	0.399
Complementary therapy, n (%)				
EVD placement	61 (13%)	37 (13%)	24 (13%)	0.835
Lumbar drainage	90 (19%)	50 (17%)	40 (22%)	0.172
Complications, *n* (%)				
SRCI	145(31%)	92 (32%)	53 (29%)	0.637
DCI	85 (18%)	63 (22%)	22 (12%)	0.010*
Urinary tract infection	35 (7%)	28 (10%)	7 (4%)	0.022*
Pulmonary infection	204 (43%)	118 (40%)	86 (48%)	0.117
Intracranial infection	13 (3%)	8 (3%)	5 (3%)	1
Bacteremia	8 (2%)	5 (2%)	5 (3%)	0.651
Venous thrombosis	53 (11%)	27 (9%)	26 (14%)	0.082
Jugular veins	36 (8%)	18 (6%)	18 (10%)	
Upper extremities	7 (1%)	3 (1%)	4 (2%)	
Lower extremities	10 (2%)	9 (3%)	1 (1%)	
Airway management, *n* (%)				
Emergency intubation	64 (14%)	32 (11%)	32 (18%)	0.036*
Preoperative	35 (7%)	14 (5%)	21 (12%)	
Postoperative	30 (6%)	18 (6%)	12 (7%)	
Tracheotomy	77 (16%)	38 (13%)	39 (22%)	0.013*
Length of stay, days, mean ± SD	23.1 ± 17.2	22.8 ± 16.3	23.7 ± 18.5	0.551
Outcome at discharge				
GCS, median (IQR)	15(15–15)	15(15–15)	15(12–15)	0.492
mRS, median (IQR)	1(1–4)	1(1–4)	1(1–4)	0.331
mRS ≤ 2, *n* (%)	323(68%)	204(70%)	119(66%)	0.394

*ACA* anterior cerebral artery, *ACoA* anterior communicating artery, *C6* ophthalmic segment of ICA, *C7* posterior communicating segment of ICA, *DCI* delayed cerebral ischemia, *EVD* external ventricular drainage, *GCS* Glasgow Coma Scale, *ICA* internal carotid artery, *IQR* interquartile range, *MCA* middle cerebral artery, *mRS* modified Rankin Scale, *NA* not applicable, *PC* posterior circulation, *SRCI* surgery-related cerebral infarction, *WFNS* World Federation of Neurological Surgeons

^*^p < 0.05

### Treatment and Outcome

Women were more likely to receive endovascular treatment (67% vs. 59%, *p* = 0.059) and had a higher rate of stent use (84% vs. 76%, *p* = 0.015). The incidence of intraoperative rerupture in our cohort was 3% (13 of 472), and the rate of EVD use was 13% (61 of 472), with no sex differences in either metric. Among postoperative complications, the incidence of DCI was significantly higher in women (22% vs. 12%, *p* = 0.01), and urinary tract infections were also significantly higher (10% vs. 4%, *p* = 0.022) in women. Men were more likely to experience emergency intubation (*p* = 0.036) and tracheotomy (*p* = 0.013), with one man undergoing both preoperative and postoperative intubation. There was no difference in the incidence of SRCI, pulmonary infection, venous thrombosis, bacteremia, and intracranial infections in women compared with men. No sex differences were found in the GCS score or in the percentage of patients achieving functional independence at discharge.

### Risk Factors for Poor Prognosis

Factors with a *p* value < 0.1 in the univariate analysis (Table 2) of poor prognosis were included in the multivariable analysis. After adjusting for age, the multivariable regression analysis (Table 3) showed that hypertension (odds ratio [OR] 2.139, 95% confidence interval [CI] 1.027–4.457), preoperative rerupture (OR 12.240, 95% CI 1.491–100.458), pulmonary infection (OR 2.297, 95% CI 1.070–4.930), EVD placement (OR 4.382, 95% CI 1.550–12.390), bacteremia (OR 14.943, 95% CI 1.412–158.117), SRCI (OR 8.588, 95% CI 4.092–18.023), venous thrombosis (OR 5.283, 95% CI 1.859–15.013), higher modified Fisher grade (*p* = 0.003) and Hunt–Hess grades (*p* = 0.035) were associated with poor prognosis, whereas the results indicated that DCI (OR 1.394, 95% CI 0.591–3.292) was not an independent risk factor for poor prognosis. When sex was forced into the regression model (Table 4), women (OR 1.768, 95% CI 0.619–5.051) were not found to be associated with poor prognosis at discharge.

**Table 2:** Univariate analysis of poor prognosis at discharge

Variable	mRS > 2 (*n* = 149)	mRS ≤ 2 (*n* = 323)	*P* value
Women, *n* (%)	88 (59%)	204 (63%)	0.394
Age, y, median (IQR)	60 (53–63)	53 (47–61)	< 0.001*
Hypertension, *n* (%)	88 (59%)	154 (48%)	0.021*
Diabetes, *n* (%)	11 (7%)	16 (5%)	0.291
Smoker, *n* (%)	45 (30%)	72 (22%)	0.064
Drinker, *n* (%)	34 (23%)	55 (17%)	0.135
Hyperlipidemia, *n* (%)	46/120 (38%)	143/291 (49%)	0.046*
Modified Fisher, median (IQR)	4 (2–4)	2 (2–2)	< 0.001*
Hunt–Hess, median (IQR)	3 (2–4)	1 (1–2)	< 0.001*
Multiple aneurysms, *n* (%)	32 (21%)	101 (31%)	0.028*
Intracerebral hematoma, *n* (%)	61 (41%)	41 (13%)	< 0.001*
Preoperative rerupture, *n* (%)	18 (12%)	2 (1%)	< 0.001*
Intraoperative rerupture, *n* (%)	6 (4%)	7 (2%)	0.398
Clip, *n* (%)	79 (53%)	90 (28%)	< 0.001*
EVD placement, *n* (%)	47 (32%)	14 (4%)	< 0.001*
Urinary tract infection, *n* (%)	11 (7%)	24 (7%)	0.985
Pulmonary infection, *n* (%)	115 (77%)	89 (28%)	< 0.001*
Intracranial infection, *n* (%)	6 (4%)	7 (2%)	0.398
Bacteremia, *n* (%)	8 (5%)	2 (1%)	0.003*
SRCI, *n* (%)	91 (61%)	54 (17%)	< 0.001*
DCI, *n* (%)	37 (25%)	48 (15%)	0.009*
Venous thrombosis, *n* (%)	39 (26%)	14 (4%)	< 0.001*

*DCI* delayed cerebral ischemia, *EVD* external ventricular drainage, *IQR* interquartile range, *mRS* modified Rankin Scale, *SRCI* surgery-related cerebral infarction

^*^p < 0.05

**Table 3:** Multivariable logistic regression analysis of poor prognosis at discharge

Potential predictors	OR	95% CI	*P* value
Age	1.043	1.005–1.081	0.025*
Hypertension	2.139	1.027–4.457	0.042*
Smoke	1.817	0.817–4.041	0.143
Hyperlipidemia	0.499	0.246–1.013	0.054
Multiple aneurysms	0.657	0.288–1.497	0.317
Intracerebral hematoma	1.378	0.552–3.440	0.493
Preoperative rerupture	12.240	1.491–100.458	0.020*
Clip	0.477	0.214–1.064	0.071
EVD placement	4.382	1.550–12.390	0.005*
Pulmonary infection	2.297	1.070–4.930	0.033*
Bacteremia	14.943	1.412–158.117	0.025*
SRCI	8.588	4.092–18.023	< 0.001*
DCI	1.394	0.591–3.292	0.448
Venous thrombosis	5.283	1.859–15.013	0.002*
Modified Fisher			0.003*
1	Reference		
2	3.530	0.627–19.874	0.153
3	29.138	2.932–289.564	0.004*
4	12.657	1.912–83.788	0.008*
Hunt–Hess			0.035*
1	Reference		
2	1.177	0.478–2.896	0.723
3	2.112	0.796–5.603	0.133
4	5.381	1.498–19.326	0.010*
5	9.045	1.264–64.741	0.028*

*CI* confidence interval, *DCI* delayed cerebral ischemia, *EVD* external ventricular drainage, *OR* odds ratio, *SRCI* surgery-related cerebral infarction

^*^p < 0.05

**Table 4:** Multivariable logistic regression analysis of poor prognosis at discharge (including sex)

Potential predictors	OR	95% CI	*P* value
Sex (women)	1.768	0.619–5.051	0.288
Age	1.040	1.002–1.079	0.036*
Hypertension	2.208	1.048–4.649	0.037*
Smoke	2.825	0.895–8.919	0.077
Hyperlipidemia	0.478	0.233–0.978	0.043*
Multiple aneurysms	0.646	0.285–1.466	0.296
Intracerebral hematoma	1.471	0.582–3.718	0.415
Preoperative rerupture	10.845	1.314–89.535	0.027*
Clip	0.464	0.206–1.048	0.065
EVD placement	4.351	1.526–12.401	0.006*
Pulmonary infection	2.387	1.108–5.144	0.026*
Bacteremia	13.917	1.334–145.241	0.028*
SRCI	8.472	4.020–17.852	< 0.001*
DCI	1.376	0.585–3.233	0.464
Venous thrombosis	4.982	1.751–14.170	0.003*
Modified Fisher			0.003*
1	Reference		
2	3.492	0.619–19.703	0.157
3	27.259	2.698–275.393	0.005*
4	12.775	1.921–84.937	0.008*
Hunt–Hess			0.028*
1	Reference		
2	1.237	0.501–3.059	0.645
3	2.177	0.819–5.782	0.119
4	6.232	1.656–23.453	0.007*
5	9.932	1.336–73.838	0.025*

*CI* confidence interval, *DCI* delayed cerebral ischemia, *EVD* external ventricular drainage, *OR* odds ratio, *SRCI* surgery-related cerebral infarction

^*^p < 0.05

### DCI and SRCI

In Table 5, we further compared the clinical characteristics and impact on outcome of DCI and SRCI. Approximately 80% (68 of 85) of DCIs could be observed on imaging as cerebral infarct lesions. Nineteen percent of DCIs and 10% of SRCIs presented as asymptomatic infarctions, and 27% of DCIs and 20% of SRCIs could not be clinically assessed because of impairment of consciousness already present in the patients due to other reasons. The proportion of symptomatic patients was significantly higher in the SRCI group than in the DCI group (70% vs. 54%, *p* = 0.018), and the distribution of clinical symptoms differed between the two ischemia types. Deterioration of consciousness and hemiparesis were more prevalent in the SRCI group, accounting for 43% and 55%, respectively, compared with only 22% and 27% in the DCI group. The incidence of aphasia was as high as 20% in the DCI group but only 10% in the SRCI group. Two cases of oculomotor nerve palsy were due to midbrain infarction caused by DCI rather than the occupying effect of the aneurysm. The proportion of patients who fully recovered from cerebral ischemia was higher in the DCI group (*p* < 0.001) compared with the SRCI group, and more patients were discharged with mRS > 2 in the SRCI group (*p* = 0.005). Neither patients with DCI nor patients with SRCI showed sex differences in discharge outcomes.

**Table 5:** Characteristics of different ischemic types

	DCI				SRCI				*P* value
	All (*n* = 85)	Women (*n* = 63)	Men (*n* = 22)	*p* value^a^	All (*n* = 145)	Women (*n* = 92)	Men (*n* = 53)	*p* value^a^	
Visible lesion on imaging, *n* (%)	68 (80)	49 (78)	19 (86)	0.577	(100)	(100)	(100)	…	…
Clinical manifestation, *n* (%)									
Asymptomatic infarction	16 (19)	13 (21)	3 (14)	0.685	15 (10)	11 (12)	4 (8)	0.401	0.069
Not assessable	23 (27)	16 (25)	7 (32)	0.559	29 (20)	14 (15)	15 (28)	0.058	0.217
Symptomatic	46 (54)	34 (54)	12 (55)	0.963	101 (70)	67 (73)	34 (64)	0.274	0.018*
Later infarction in symptomatic DCI	29 (63)	20 (59)	9 (75)	0.516	NA				…
Classification of symptoms, *n* (%)									
Deterioration of consciousness	19 (22)	11 (17)	8 (36)	…	62 (43)	42 (46)	20 (38)	…	…
Hemiparesis	23 (27)	15 (24)	8 (36)	…	80 (55)	54 (59)	26 (49)	…	…
Aphasia	17 (20)	14 (22)	3 (14)	…	14 (10)	8 (9)	6 (11)	…	…
Encephalopathy	3 (4)	3 (5)	0	…	8(6)	5 (5)	3 (6)	…	…
Facial paralysis	2 (2)	1 (2)	1 (5)	…	0	0	0	…	…
Oculomotor nerve palsy	2 (2)	2 (3)	0	…	0	0	0	…	…
Outcome at discharge									
Complete recovery from ischemia	45 (53)	35 (56)	10 (45)	0.414	42 (29)	28 (30)	14 (26)	0.607	< 0.001*
mRS > 2	37 (44)	24 (38)	13 (59)	0.087	91 (63)	56 (61)	35 (66)	0.535	0.005*
Time of occurrence, median (IQR)									
Days after aSAH	8 (6–10)				NA				…
Days after surgery	4 (3–6)				Within 48 h				…

Encephalopathy mainly refers to a status of delirium. “Not assessable” is defined as a patient who already had impaired consciousness for other reasons and could not be clinically assessed. *p* values represent the result of the comparison between DCI and SRCI

*aSAH* aneurysmal subarachnoid hemorrhage, *DCI* delayed cerebral ischemia, *IQR* interquartile range, *mRS* modified Rankin Scale, *NA* not applicable, *SRCI* surgery-related cerebral infarction

^*^p < 0.05

^a^*p* values represent the results of the comparison between women and men

### DCI Timing

**Fig. 3:**
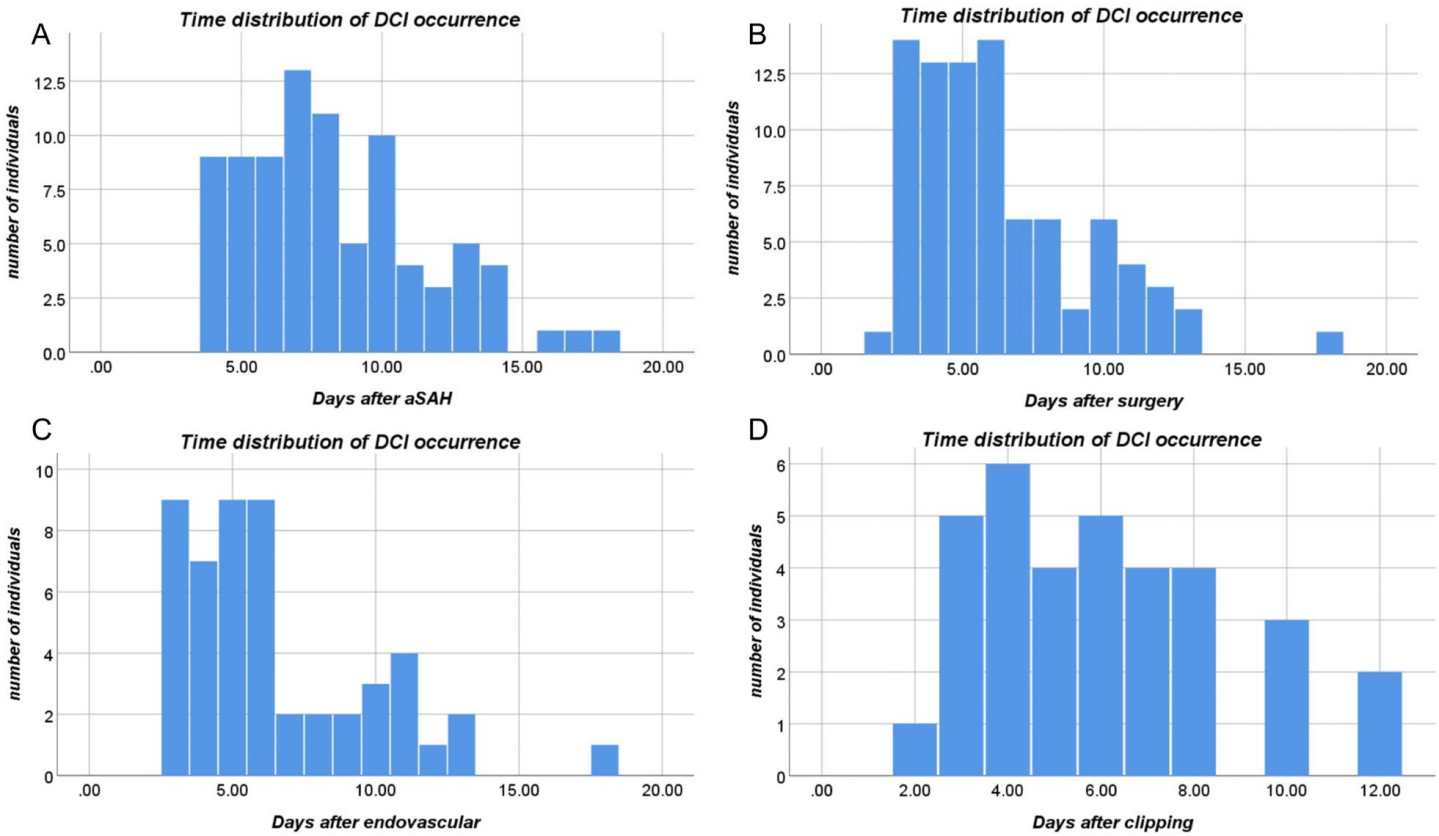
Time distribution of delayed cerebral ischemia (DCI) occurrence. For patients experiencing DCI more than once, the time was recorded based on the earliest occurrence of DCI. **a**, The period (days) based on the time after aSAH, 8 (interquartile range [IQR] 6–10) days. **b**, The period (days) based on the time after surgery (aneurysm repair), 4 (IQR 3–6). **c** and **d**, The period (days) based on the time after endovascular treatment and clipping, respectively. aSAH, aneurysmal subarachnoid hemorrhage

Figure 3 depicted the timing of DCI, in which the incidence of DCI was at a high level from day 4 to day 10 after SAH, declining to a plateau on day 11 and declining again on day 15. When using the time of surgery (aneurysm repair) as a baseline, the incidence of DCI was highest on days 3–6 postoperatively and then declined on days 7 and 11. The pattern of DCI appeared to be different between the two treatment modalities, with DCI after endovascular treatment concentrated on days 3–6, whereas the frequency of DCI after clipping declined slowly on days 3–12. Compared with clipping, DCI peaks on postoperative days 3–6 were more pronounced in endovascular treatment.

## Discussion

In this study, we comprehensively reviewed information on patients admitted to our hospital within 72 h after aSAH regarding sex differences. The majority of them (69.5%) presented with low-grade aSAH (Hunt–Hess grade ≤ 2). Women have an older age at onset than men and are more likely to be screened out for multiple aneurysms, which has been found in previous studies [10, 14, 21]. By further subdividing women according to whether they were menopausal or not, we found that perimenopause appeared to be the main time period in which women experienced aSAH. Among postmenopausal women, the number of people is greatest in the first 4 years, with a gradual decline in each subsequent 4-year period, which may be attributed to fluctuations in estrogen levels. Estrogen is involved in both aneurysm formation and aSAH [4, 22]. In a large prospective study, earlier age at menopause and shorter reproductive lifespan were found to be associated with aSAH risk in women, supporting the role of estrogen in the development of aSAH [23]. Compared with women, men had more anterior communicating artery and middle cerebral artery aneurysms and were more likely to present with intracerebral hematomas on admission, as ruptured aneurysms in these two locations usually develop hematoma [24]. Compression of brain tissue by hematoma and higher modified Fisher grades explain the worse admission status of men in our study, which was also associated with more men receiving emergency intubation (mainly preoperatively).

Multiple studies have shown a significantly higher incidence of postoperative DCI in women [10, 11, 14, 24], yet no sex differences in DCI have been found in other studies [9, 12, 13]. Such results may be attributed to different case inclusion criteria and lack of standardized DCI definition criteria. The current definition of DCI is thought to be triggered by a combination of large vessel vasospasm and multiple brain injuries induced by early brain damage after aSAH [2]. Cerebral vasospasm is thought to start on days 3–4 after aSAH and peak on days 7–10 and resolve on day 14 [25]. However, the current studies have not clearly defined the time of DCI occurrence, nor have they provided a detailed description of the clinical manifestation and outcome of DCI. Our study used a standardized definition of DCI based on previous publications [19, 20], including unexplained clinical deterioration and asymptomatic cerebral infarction within 21 days after aSAH. We found the incidence of DCI was significantly higher in women than in men, with 80% of DCIs presenting with cerebral infarct lesions. The higher incidence of DCI may be associated with a higher incidence of cerebral vasospasm in women [9, 14]. Estrogen therapy has been demonstrated to reduce vasospasm in an animal model by decreasing endothelin 1 production and increasing expression of inducible nitric oxide synthase, whereas decreased estrogen levels after menopause lead to an increased risk of vasospasm [26]. In our classification of the clinical manifestation of DCI, the incidence of symptomatic DCI (clinical deterioration triggered by DCI) was only 54%, and 27% of patients could not be assessed with symptom but were visible with cerebral infarct lesion. We also categorized DCI by symptom and calculated that more than half of DCIs were fully recovered at discharge. Time distribution plots of DCI showed that the peak of DCI was concentrated on days 4–10 after aSAH and occurred rarely after day 14, which is consistent with the time period of cerebral vasospasm and similar to the results of a previous study [25]. We attempted a new approach to describe the relationship between DCI and surgery, and the results showed a high aggregation of DCI occurrence on days 3–6 postoperatively and a decline on days 7 and 11 after surgery. Compared with clipping, DCI peaks on postoperative days 3–6 were more pronounced in endovascular treatment. The different DCI patterns we found after endovascular treatment and clipping are novel and need to be validated in larger cohorts. The proposed postoperative time window gives us a better understanding of DCI and contributes to clinical early warning.

Age, aneurysm rerupture, pulmonary infection, EVD placement, and higher Hunt–Hess and modified Fisher grades have been demonstrated to be associated with a poor prognosis after aSAH in the published literature [27–29]. Moreover, we found that hypertension, venous thrombosis, bacteremia, and SRCI were associated with poor prognosis. Indeed, venous thrombosis is a consequence of the patient’s impaired physical mobility and the hypercoagulable state of the blood rather than a predictor. Among venous thrombotic events, a high incidence of jugular vein thrombosis was associated with the use of central venous catheters. The absence of prophylactic use of heparin in our institution increased this rate somewhat given the risk of bleeding. Hypertension is a risk factor for aneurysm growth and rupture [30]. The hypertensive patients in this study had higher modified Fisher grades on admission, and more bleeding may have contributed to a poor outcome. Early cerebral infarction was used in some articles to describe cerebral infarction at 24 or 24–48 h postoperatively [28, 31]. Early cerebral infarction had a greater impact on poor prognosis at 3 months compared with cerebral infarction after 24 h of surgery [28], and cerebral infarction within 24–48 h after surgery was also found to be a strong predictor of poor prognosis in another study, whereas delayed cerebral infarction was not [31]. Interestingly, surgical procedure was found to be an independent risk factor for early cerebral infarction in both of these studies. SRCI is also the main cerebral ischemic event that needs to be differentiated from DCI, and reporting both types of ischemic events can help us to distinguish the prognostic implications of different ischemia types. In this study, SRCI was defined as clinical deterioration occurring early (within 24–48 h postoperatively) after excluding other causes, and new infarction could be detected by early CT examination. The majority of symptomatic SRCIs in our study occurred immediately after surgery and could be inferred to be related to surgery from surgical records or postoperative CT, and multivariable regression suggests SRCI to be an independent risk factor for poor prognosis.

It is worth noting that DCI was not an independent risk factor for poor prognosis in our regression model, which is consistent with a previous study [31]. Because DCI presents with diverse symptoms, the rate of alleviation varies by symptom type. Fourteen of 17 patients with aphasia in the DCI group in our study recovered completely at discharge, whereas only 8 of 23 recovered completely from hemiparesis, and it is even more important to detect and start treatment early. Both DCI and SRCI are important types of cerebral ischemia after aneurysm repair, but there are some differences between them. Our results demonstrate that SRCI is more likely to result in symptomatic presentation and lower remission rates at discharge compared with DCI, which provides another indication that stroke related to the surgical procedure is associated with less recovery than DCI-related deficits. Assuming that DCI is caused by vasospasm, early intervention might reverse this neurologic dysfunction, yet not all DCIs are associated with vasospasm [32]. SRCI is a direct result of surgery involving large vessel occlusion, inadequate tissue perfusion, and surgical trauma. As a result, recovery from neurologic dysfunction is more difficult. Finally, we found that sex differences did not affect the outcome of aSAH, which is consistent with another study [16]. Despite the higher incidence of DCI in women, the overall higher remission rate of DCI weakened the impact of sex differences on outcomes.

There are some limitations to this article, and the results should be carefully and reasonably interpreted. The relationship between menstrual history and aSAH in women was included in our study, and we were unable to predict the age at menopause in premenopausal women but only made reasonable assumptions about the risk of aSAH in this population through the age distribution. Menstrual history was missing for some patients because the data could not be obtained from unconscious patients or their families at the time of admission, which may have an impact on the accuracy of the results. We used the mRS at discharge rather than the long-term outcome to assess prognosis, which influenced the weighting of some risk factors. Moreover, the definition of DCI in this study included both imaging and clinical symptoms, which may have increased the incidence of DCI to some extent. Evidence regarding the high incidence of DCI in women remains to be validated by transcranial Doppler ultrasound and additional evidence of vascular reactivity. Because review was done by CT in most cases and magnetic resonance imaging evaluation after DCI episodes was lacking, the distinction between aphasia (focal lesions in the dominant hemisphere) and global encephalopathy that may result in impaired speech output was limited.

## Conclusions

Women have a higher incidence of DCI, but there is no sex difference in outcomes after aSAH, and poor prognosis is associated with worse admission condition and perioperative complications. SRCI is a strong independent risk factor for poor prognosis, whereas DCI is not.
